# Interaction between plants and epiphytic lactic acid bacteria that affect plant silage fermentation

**DOI:** 10.3389/fmicb.2023.1164904

**Published:** 2023-06-06

**Authors:** Lijuan Chen, Yili Wang, Xi Li, Jennifer W. MacAdam, Yunhua Zhang

**Affiliations:** ^1^College of Animal Science and Technology, Anhui Agricultural University, Hefei, China; ^2^College of Agriculture and Applied Sciences, Utah State University, Logan, UT, United States; ^3^College of Resources and Environment, Anhui Agricultural University, Hefei, China

**Keywords:** plant, epiphytic, lactic acid bacteria, interaction, silage

## Abstract

Lactic acid bacteria (LAB) have the ability to ferment water-soluble carbohydrates, resulting in the production of significant amounts of lactic acid. When utilized as additives in silage fermentation and feed, they have been shown to enhance the quality of these products. Epiphytic LAB of plants play a major role in the fermentation of silage plants. Plant species in turn affect the community structure of epiphytic LAB. In recent years, an increasing number of studies have suggested that epiphytic LAB are more effective than exogenous LAB when applied to silage. Inoculating silage plants with epiphytic LAB has attracted extensive attention because of the potential to improve the fermentation quality of silages. This review discusses the interaction of epiphytic LAB with plants during silage fermentation and compares the effects of exogenous and epiphytic LAB on plant fermentation. Overall, this review provides insight into the potential benefits of using epiphytic LAB as an inoculant and proposes a theoretical basis for improving silage quality.

## 1. Introduction

Ensiling is a fermentation process during which lactic acid bacteria (LAB) convert water-soluble carbohydrates into organic acids under anaerobic conditions. This method is effective in developing sustainable feed sources (Xie et al., 2021) that would otherwise go to waste in food production, such as corn stalks and sorghum stalks, which are the main feed sources for ruminants in developing countries (Yanti et al., 2019). Prior to the development of LAB inoculants, forage plants hosted an unpredictable number of epiphytic LAB, making it difficult to meet the requirement for successful silage. Inoculants of LAB are used to improve silage quality (Pholsen et al., 2016; Peng et al., 2021).

LAB refers to low G + C, Gram-positive bacteria (Holzapfel and Wood, 2014). They play a vital role in the production of more than 3,500 different types of fermented products, such as silages and fermented foods (Tamang et al., 2016). LAB-dependent fermentation improves the nutritional value, sensory properties, and safety of plants (Marco et al., 2017). LAB can tolerate low pH and have excellent acid production ability. Adding LAB to silage reduces the pH value, increases lactic acid (LA) content, and the number of LAB during the ensiling process while competitively inhibiting harmful bacteria (Wang et al., 2021). The effects of epiphytic LAB on silage are superior to those of exogenous LAB from inoculants (Wang et al., 2018; Cheng et al., 2022). This review explores the interactions between epiphytic LAB and host plants, primarily considering silage plants, as well as the reasons for these interactions. The aim of this review is to provide guidance for the subsequent selection of epiphytic LAB as inoculants to improve the effectiveness of the ensiling process.

## 2. Interaction between plants and their epiphytic LAB

### 2.1. Types of plant epiphytic LAB

Many kinds of epiphytic microorganisms are associated with plants, and the species and quantity of epiphytic microorganisms are affected by this association (Knief et al., 2010; Vogel et al., 2016). Species type, seasonal variation and geographical location are all regulating factors for the composition of the plant epiphytic bacterial community (Maignien et al., 2014). It has been suggested that neighboring plants may share a similar community composition of epiphytic microorganisms due to their proximity, as they serve as potential inoculum sources for these microorganisms (Bulgarelli et al., 2013). Additionally, some studies have indicated that plants may recruit microorganisms to counteract pathogens (Vandenkoornhuyse et al., 2015). Furthermore, drought and low-temperature stress can significantly affect the diversity and abundance of plant-associated microbial communities, including LAB (Fitzpatrick et al., 2018; Fabiszewska et al., 2019).

Epiphytic LAB can be defined by microbial culturing and identification techniques. Several techniques have been employed to study the bacterial community associated with plants, including culture-based methods such as most probable number, selective medium, and biochemical analysis, as well as non-culture-based methods such as denaturing gradient gel electrophoresis, single-strand conformational polymorphism, and terminal restriction fragment length polymorphism (T-RFLP), or a combination of these methods (McAllister et al., 2018). In recent years, 16S rRNA gene sequencing has been widely used to detect epiphytic LAB (as shown in Supplementary Table S1), often in combination with culture methods. The following were discovered using the combination of culture and 16S rRNA gene sequencing method: *Lactiplantobacillus plantarum* was the main epiphytic LAB in plants as diverse as hybrid elephant grass, alfalfa, and black tea, *Pediococcus pentosaceus* was the main epiphytic LAB of oat in the Qinghai–Tibet Plateau and *Weissella* in wormwood (Chaikaew et al., 2017; Nascimento Agarussi et al., 2019; Yu et al., 2020; dos Santos Leandro et al., 2021). *Enterococcus faecium* was the main epiphytic LAB of sorghum (Rena et al., 2012).

LAB can be classified into homofermentative and heterofermentative types based on their metabolism. Homofermentative LAB use glycolysis to convert glucose into two molecules of pyruvate, which is then converted into LA. Heterofermentative LAB are commonly used as silage inoculants, and today, most of the bacteria in this group are considered facultative heterofermentative LAB. Facultative heterofermentative LAB have phosphoketolase, which allows them to primarily ferment pentose to produce LA (Pahlow et al., 2003; Muck et al., 2018). Heterofermentative LAB, now called obligate heterofermentative LAB, can convert glucose into LA, ethanol as well as carbon dioxide, and produce other metabolites such as acetic acid and ethanol (Muck et al., 2018; Benjamim da Silva et al., 2021). Some of the commonly found epiphytic LAB on plants include *L. plantarum*, *Lentilactobacillus buchneri, E. faecium*, and *Pediococcus acidilactici*. Additionally, recent studies have identified other species of plant epiphytic LAB such as *Weissella kimchii*, *Enterococcus flavescens*, *Lactobacillus taiwanensis*, *Leuconostoc lactis*, *Enterococcus mundtii*, as well as *Weissella cibaria*, and the presence of these LAB plays an important role in improving the quality of plant silage (Brusetti et al., 2006; Wang et al., 2009; Pang et al., 2011; Fabiszewska et al., 2019).

### 2.2. Influences of plants on their epiphytic LAB

The environment of plants is often challenging for epiphytic microorganisms. The concentration of nutrients in the leaf layer, water availability, ultraviolet radiation, oxidative stress, and temperature changes affect microbial growth (Yu et al., 2020); The waxy cuticle on plant leaves interfered with the colonization of plant microorganisms by restricting the diffusion of nutrients from the plant interior to the surface and reducing surface wetness of the leaves (Lindow and Brandl, 2003). However, epiphytic microorganisms can themselves reduce the effects of ultraviolet radiation on plants through pigmentation. The epiphytic LAB of leaves are affected by leaf vein, hair, stomatal, and other leaf structures. LAB on the leaf surface are easily washed away by rain or killed by peroxide and ultraviolet light, whereas the LAB in the stomata survive relatively easily (Lindow and Brandl, 2003).

Plant roots are the main gathering location of microorganisms (including LAB). Strafella et al. (2020) demonstrated that the rhizosphere of plants was rich in nutrients released by root secretions, thereby creating a suitable ecological niche for the proliferation of microorganisms. The shedding of root cells and the release of mucilage deposit large amounts of material into the rhizosphere, including plant cell wall polymers such as cellulose and pectin, which induce microbial aggregation to use it as a carbon source (Turner et al., 2013). The overlap between the bacteria on the roots and those attached to the woody structure suggests that the rhizosphere provides attachable structures for microorganisms, and that plant root LAB can spread to other parts of the plant through this overlapping area. The role of plant roots in interacting with epiphytic LAB, has been relatively understudied and is an area to be considered for future research.

Many secondary plant metabolites, such as tannins, anthocyanins, lignin, and alkaloids, may possess antibiotic properties affecting LAB. Catechin has a positive effect on the growth of oxygen-sensitive probiotics (such as *Lactobacillus helveticus*) (Gaudreau et al., 2012). Zhu H. et al. (2022) pointed out that polyphenols, polysaccharides, and saponins in plants can promote the growth and metabolism of LAB. The phenolic compounds in olive fruits can decrease bacterial growth (Rodríguez et al., 2009). Plant anthocyanins could bind to the phospholipid bilayer in the cell membrane of a microorganism, which could damage the cell membrane. Na et al. (2020) showed the inhibitory effects of anthocyanins on LAB and damage to the cell protein of LAB. Tannic acid inhibited LAB growth by affecting metabolic enzymes through tannin–protein interaction (Bossi et al., 2007).

LAB can successfully colonize intact plants. Glucose, fructose, and sucrose are the preferred carbon sources for LAB growth and fermentation, and these sugars are the main sugars found in the leaf layer (Lindow and Brandl, 2003). LAB use sugar inside plants by entering the plant interior through open places such as stomata and wounds on plant leaves (Gnanamanickam and Immanuel, 2007).

The aforementioned studies emphasize that the intrinsic and extrinsic environments of the plant, as well as plant compounds and secondary metabolites (e.g., tannins and lignin) affect LAB.

### 2.3. Effects of plant epiphytic LAB on host plants

As a type of beneficial bacteria, LAB can influence plant growth.

#### 2.3.1. Direct or indirect degradation of plant compounds

Epiphytic LAB can degrade plant compounds directly or indirectly, thereby affecting the plant itself. Plant epiphytic LAB can decompose complex plant polysaccharides (e.g., hemicellulose) and soluble sugars (e.g., galactose, arabinose) (Yu et al., 2020). The presence of plant phenolics during microbial activity drives the evolution of microorganisms in favor of their own survival. *L. plantarum* could degrade phenolic compounds through methods such as lowering pH and producing organic acids (Rodríguez et al., 2009). Furthermore, low concentrations of phenolics stimulated LAB growth, whereas high concentrations of phenolics could disrupt microbial cell integrity and delay LAB metabolism of carbohydrates (Filannino et al., 2018). Low pH value is also conducive to the degradation of phytate, and LAB can reduce pH by producing organic acids to promote phytate degradation, and provide favorable conditions for the endogenous cereal phytase activity (Reale et al., 2007).

#### 2.3.2. Production of compounds that directly affect plant metabolism

LAB are capable of producing various compounds, such as lactic acid, acetic acid, and hydrogen peroxide, which are strongly associated with the defense, growth, and development of the organism and signal transduction, and these compounds will have an impact on plants (Konappa et al., 2016; Wink, 2016). LAB acidification can reduce post-harvest decay caused by pathogens and inhibit the production of mold toxins (Oliveira et al., 2014). Additionally, LAB can facilitate tissue repair in damaged plants and enhance their immune response and disease resistance (Raman et al., 2022). LAB can be use as bioprotective agents, it can produce plant growth-promoting hormones (e.g., indoleacetic acid and gibberellin) that can enhance plant growth (Abhyankar et al., 2021), it also induce the production of defense-related enzymes (phenylalanine ammonialyase, polyphenol oxidase, peroxidase and β-1,3-glucanase) to resist bacterial wilt caused by *Ralstonia solanacearum* (Murthy et al., 2012; Konappa et al., 2016). *Verticillium dahliae* is a fungus that can cause Verticillium wilt disease, which affects crop growth and leads to a significant decrease in crop yield. However, the Enterococcus strain can inhibit this fungus (Fhoula et al., 2013). In addition to the main metabolites, the secondary metabolites of LAB [e.g., bacteritin and exopolysaccharides (EPS)] also play a significant role (Fernandes and Jobby, 2022).

##### 2.3.2.1. Bacteriocin

Bacteriocins secreted by LAB are a family of ribosomally synthesized antimicrobial peptides (AMP) with a wide activity against bacteria and fungi. Various LAB could produce different bacteriocins, and *Lactococcus* mainly produces class I bacteriocins, such as nisin. *L. plantarum* can produce at least six different bacteriocins (e.g., plantaricin). *Pediococcus* produce class II bacteriocins, such as pediocin A (Fernandes and Jobby, 2022). Plant pathogens and toxins can cause severe diseases in plants, leading to growth inhibition (Evidente, 2019). Bacteriocins can inhibit pathogens and toxins in plant. Such as, Nisin produced by LAB can inhibit the growth of *Pseudomonas aeruginosa* and cause cellular damage or death (Mazzotta et al., 1997; Ghapanvari et al., 2022) while Lactocidin and nisin have inhibitory effects on *Xanthomonas campestris*, *Erwinia carotovora*, and *Pseudomonas syringae* (Visser et al., 1986). When combined with EDTA, nisin shows enhanced antimicrobial activity against Gram-positive and some Gram-negative pathogens, such as *X. campestris*, *E. carotovora* (Wells et al., 1998; Belfiore et al., 2007). Bacteriocins mainly act as bacteriostatic agents that reduce infection, decay, and death of host plants by inhibiting the growth of harmful bacteria. Interestingly, LAB can use the autoimmune protein system to protect itself from being killed by the bacteriocin that it produces while producing bacteriocin to destroy other microorganisms (Oppegård et al., 2007).

##### 2.3.2.2. Exopolysaccharides

EPS produce by LAB play a crucial role in plant physiology (Fernandes and Jobby, 2022). EPS refers to biopolymers secreted outside of cells, which is divided into homopolysaccharide and heteropolysaccharide during the growth of microorganisms (Nwodo et al., 2012). EPS has a drought-resistant effect and can ensure the growth of plants under drought stress (Costa et al., 2018). In addition, EPS has other biological functions, such as antioxidant, antibacterial, immunomodulatory, and even antiviral effects (Zhou et al., 2019); this all contributes to the growth of plants. Hydroxyl free radicals and singlet oxygen are reactive oxygen species (ROS) that have oxidative capacity that is harmful to aerobic organisms, and superoxide can trigger a series of reactions, thereby producing hydroxyl free radicals and other destructive substances (Waszczak et al., 2018). EPS secreted by LAB exhibits antioxidant properties by scavenging superoxide anion and DPPH free radicals and can also sorb heavy metals in the environment through the presence of functional groups (Zhang et al., 2020). Jiang et al. (2018) stated that strains that could produce EPS exhibited antibacterial activity and cold resistance. Bacterial spot disease can cause huge losses in tomato production, spraying EPS on tomato leaves can control bacterial spot disease and stimulate the defense mechanism of plants (Blainski et al., 2018). After being attacked by pathogens, plants treated with EPS can be protected against extracellular pathogens by improving the cellular activities of polyphenol oxidase (PPO), catalase (CAT), and SOD and by accelerating the accumulation of cellulosic compounds on the inner cell wall surface. Microbial or pathogen-associated molecular patterns (MAMP/PAMP) are the basic structures maintained in pathogenic, non-pathogenic, and saprophytic microorganisms. MAMP/PAMP can be rapidly recognized by receptors on the plant cell surface to induce different defense responses. EPS can act as MAMP to induce resistance in plants (Jones and Dangl, 2006; Blainski et al., 2018). LAB produced EPS on plants can induce stomatal closure. These mechanisms, along with the activation of antioxidant enzymes in planta, help reduce disease severity in plants because they impede bacterial infection and colonization (Melotto et al., 2006). Therefore, LAB can promote plant growth metabolism and inhibit pathogen survival by producing various compounds directly and indirectly during plant growth.

## 3. Effect of LAB on silage fermentation quality

### 3.1. Effects of LAB inoculation on feed silage fermentation quality

The inoculation of silage fermentation plants with LAB is currently one of the main approaches to improving the fermentation quality of silage. LAB inoculants can enhance the abundance of beneficial bacteria and suppress harmful microorganisms while accelerating the production of LA, thereby improving nutritional quality as well as fermentation characteristics and decreasing the harmful microorganism composition of silage (Ávila et al., 2014; Bai et al., 2021). Biogenic amines that are produced by plants during fermentation or protein degradation are organic compounds widely present in plants. Excessive intake of biogenic amines may cause poisoning in humans and animals (Wójcik et al., 2021). A type of laccase in LAB can oxidize and degrade biogenic amines in plants (Callejón et al., 2016; Xu et al., 2022). Nitrite is toxic to plants, and high concentrations of nitrite can affect plant growth and development. LAB can facilitate nitrite degradation by lowering the pH value (Wu et al., 2015). Meanwhile, plant metabolites can also affect the fermentation process. During the fermentation process of plants, harmful microorganisms such as *Escherichia coli* and *Salmonella* may be produced, lactic acid and bacteriocins can inhibit the growth of these microorganisms (Queiroz et al., 2018; Lu et al., 2020; Yan et al., 2021). EPS can affect the viscosity, dehydration, and sensory properties which may prolong shelf life of silage (Zhou et al., 2019). Inoculation of native grass with *L. plantarum* significantly improved the nutritional characteristics and fermentation quality of the ensiled feed. Specifically, the content of crude protein, carbohydrates, and LA increased, while the contents of propionic acid and ammonia nitrogen decreased compared to the control group (Li et al., 2022). Additionally, the LAB inoculation increased the abundance of LAB and decreased the abundance of aerobic bacteria, yeast, and coliform bacteria. Bai et al. (2021) used *L. plantarum*, *P. pentosaceus*, and *Enterococcus faecalis* to ensile alfalfa, and the results showed that LAB inoculation could reduce the pH of silage, increase the production of lactate, and change the composition of the bacterial community. Inoculating corn silage with *L. plantarum* and *L. buchneri* reduced *E. coli* and improved silage quality (Li et al., 2021). Ensiling *Stylosanthes guianensis* and whole-plant soybean with *L. plantarum* improved feed fermentation quality and reduced protein decomposition (Gao et al., 2022). Similarly, LAB inoculation of paper mulberry silage reduced pH, increased organic acid content, improved nutrient composition, and enhanced aerobic stability (Zhang et al., 2022). The addition of specific LAB during fermentation promoted their growth (Bao et al., 2016). For instance, Xu et al. (2017) used *Levilactobacillus brevis* and *Lentilactobacillus parafarraginis* to ferment corn straw, and found that *Lactobacillus* became more abundant after fermentation, while *Lactococcus* decreased during ensiling. Bao et al. (2016) added *P.acidilactici* and *L. plantarum* to silage alfalfa and found that the added LAB became the dominant bacterial species in the late stages of ensiling.

The amount of LAB inoculant is an important factor affecting the fermentation quality of silage. Harrison et al. (1989) reported that at least 10^5^ CFU/g of LAB were needed to ensure a significant improvement in silage quality. In their study, grass-legume forage was inoculated with 10^5^ CFU/g and 10^6^ CFU/g of LAB, resulting in an increase in the quantity and nutritional value of LAB in forage compared with the control group, with the higher dosage having a better effect. The inoculation of 10^7^ CFU/g fresh mass *L. plantarum* in alfalfa had a better effect than 10^5^ CFU/g, this may be related to the buffering capacity of alfalfa, which accumulates higher concentrations of calcium than grass forages (Zhu Y. et al., 2022). Inoculation of *L. buchneri* could improve the aerobic stability of silage, and a dosage higher than 10^5^ CFU/g fresh mass was more effective (Kleinschmit and Kung, 2006). Therefore, the amount of LAB inoculant should be formulated to achieve optimal fermentation quality for different silage plant species.

### 3.2. Effects of epiphytic LAB inoculation on feed silage fermentation quality

Two reasons account for the better efficacy of inoculating epiphytic LAB in silage feed. First, the differences in the preference of different LAB species for the utilization of carbohydrates from various raw materials (Wang et al., 2018). For example, *L. plantarum* can adapt to various environments and carbohydrates; *Latilactobacillus sakei* can utilize a wider range of carbohydrates compared with *Weissella* and *Leuconostoc* in kimchi (Gustaw et al., 2021); *L. plantarum* demonstrates a strong ability to utilize glucose and fructose, while *Lactobacillus acidophilus* exhibits a stronger capacity to utilize lactose and maltose (Xie, 2021). Second, LAB of the same species exhibited adaptive preferences that were shaped by their living environment, the types of carbohydrates available in various raw materials and various metabolites. Liu (2021) pointed out that strains undergo genetic evolution influenced by environment, leading to the emergence of unique genotypes, which confer distinct phenotypes and physiological functions to these strains. For example, the strains associated with plant ecological niches encode a broader metabolic pathway than those of dairy strains, because lactose is the main carbon source in milk, whereas each plant ecological niche has a separate carbohydrate composition. Thus, *L. lactis* in plants can metabolize various plant carbohydrates, whereas *L. lactis* in dairy products do not. Meanwhile, two *L. lactis*, namely, KF147 and A12, from different plant environments, metabolize raffinose in two different ways due to the different genes involved in the metabolism of raffinose (Laroute et al., 2017). The different sources of *L. plantarum* have unique physiological and biochemical traits and will produce different effects when applied to silage (Cheng et al., 2022). For example, *L. plantarum* from *Phalaris arundinacea* silage has positive glucose gas production, whereas the *L. plantarum* strains isolated from pickles showed negative gas production. Conversely, *L. plantarum* strains isolated from *P. arundinacea* displayed better tolerance to low temperature and acidic conditions than those from pickles, but showed lower tolerance to high temperature than the pickle strains. In the realm of plant science, the specific type and concentration of tannins present in plants can exert distinct inhibitory effects on various strains of LAB (Dong et al., 2019). Research has shown that low concentrations of tannins may serve to stimulate the growth of LAB, while higher concentrations of tannins can serve to impede the growth of LAB. Inhibitory tests using extracted tannins against LAB were carried out by Vivas et al. (2000) who found that both ellagitannins extracted from oak wood (hydrolysable tannins) and procyanadins extracted from grape seed (condensed tannins) acted as bacterial growth inhibitors; *Oenococcus oeni* survived better in grape tannins. The authors concluded that this grape epiphytic LAB may tolerate secondary metabolites from grapes better than secondary metabolites from oak wine casks.

Compared with exogenous LAB, epiphytic LAB have stronger growth and acid production capabilities during fermentation, which can cause faster pH reduction, and more significant carbohydrate consumption ability. In addition, the fermentation of epiphytic LAB results in higher levels of ascorbic acid, glutathione, and total antioxidant activity, which can prolong the shelf life (Di Cagno et al., 2013). When either exogenous or native epiphytic *L. buchneri* were used to ensile whole corn under laboratory and field conditions, the native strains had higher aerobic stability (Carvalho et al., 2021). The quality improvement of fermentation after adding epiphytic LAB was better than using commercial LAB (Wang et al., 2009). Cheng et al. (2022) used epiphytic and exogenous *L. plantarum* to ensile mulberry leaves, and the results showed that the epiphytic LAB inoculant resulted in improved silage quality. The commercial culture of *L. plantarum* had some antagonism with the natural epiphytic bacteria of a particular barley, thereby resulting in higher protein loss during ensiling (Kim et al., 2015).

## 4. Conclusion

In conclusion, the intimate relationship between epiphytic LAB and host plants has been shown to facilitate their effective application in silage, surpassing that of commercial exogenous LAB. Investigating the application and mechanisms of epiphytic LAB in silage and interactions of native and exogenous LAB strains and concentrations with specific plant species will be valuable in improving silage quality and nutritional value. Future research should focus on exploring the complex interactions between epiphytic LAB and host plants, using genomic approaches to uncover the underlying reasons for the differential functional effects of various strains of LAB, elucidating the diversity of epiphytic LAB, and identifying novel application technologies for epiphytic LAB.

## Author contributions

LC, YW, XL, JM, and YZ wrote and edited the manuscript. All authors reviewed and approved the final version of the manuscript.

## Funding

This work was supported by grants from the National Natural Science Foundation of China (32172769).

## Conflict of interest

The authors declare that the research was conducted in the absence of any commercial or financial relationships that could be construed as a potential conflict of interest.

## Publisher’s note

All claims expressed in this article are solely those of the authors and do not necessarily represent those of their affiliated organizations, or those of the publisher, the editors and the reviewers. Any product that may be evaluated in this article, or claim that may be made by its manufacturer, is not guaranteed or endorsed by the publisher.
