# Supplementary material for: Interaction between plants and epiphytic lactic acid bacteria that affect plant silage fermentation
Source: Front Microbiol. 2023 Jun 6;14:1164904. doi: 10.3389/fmicb.2023.1164904 (PMC10290204; doi:10.3389/fmicb.2023.1164904)
Supplement: Supplementary file 1 [file Table_1.pdf]

Supplementary Table

Table S1 Epiphytic Lactic Acid Bacteria and Identifying Techniques

Reference	Epiphytic LAB Species	Host	Techniques
Kim et al., 2010	*Pediococcus acidilactici*	rice wine	PCR-DGGE
Ruiz Rodríguez et al., 2019	*Enterococcuscase;*<br>*Enterococcus gallinarum;*<br>*Enterococcus durans;*<br>*Enterococcus faecalis;*<br>*Enterococcus hirae;*<br>*Enterococcus mundti;*<br>*Levilactobacillus brevis;*<br>*Lacticaseibacillus rhamnosus;*<br>*Lactiplantibacillus plantarum;*<br>*Lactococcus lactis;*<br>*Lactococcus subsp. Cremoris;*<br>*Lactococcus lactis subsp. Lactis;*<br>*Weissellacibaria;*<br>*WeissellaFabalis;*<br>*Weissella minor*	wild fruits and flowers	16S rRNA gene sequencing;<br>rep-PCR
Pereira et al., 2019	*Lactiplantibacillus plantarum;*<br>*Weissella cibaria;*<br>*Weissella confuse;*<br>*Weissella paramesenteroides*	plant and cactus silage	16S rRNA gene sequencing

Kharazian et al., 2017	*Limosilactobacillus fermentum;*<br><br>*Lactiplantibacillus plantarum;*<br><br>*Lactobacillus. paralimentaris;*<br><br>*Lactiplantibacillus pentosus;*<br><br>*Lactobacillus buchneri*	corn	16S rRNA gene sequencing
Sáez et al., 2017	*Enterococcus durans;*<br><br>*Enterococcus faecium;*<br><br>*Enterococcus mundtii;*<br><br>*Enterococcus casseliflavus;*<br><br>*Lacticaseibacillus rhamnosus;*<br><br>*Lactococcus garvieae;*<br><br>*Weissellacibaria;*<br><br>*Weissellaparamesenteroides*	Common bean and Scarlet Bracket	16S rRNA gene sequencing;<br><br>rep-PCR
Eikmeyer et al., 2013	*Lactiplantibacillus plantarum;*<br><br>*Levilactobacillus brevis;*<br><br>*Lactobacillus buchneri;*<br><br>*Lactococcus lactis subsp. Lactis;*<br><br>*Leuconostoccitreum*	Grass silage	Metagenome
El et al., 2021	*Enterococcus faecium;*<br><br>*Weissella paramesenteroides;*<br><br>*Leuconostoc mesenteroides;*<br><br>*Lactiplantibacillus plantarum*	olive	16S rRNA gene sequencing
